# High-Voltage Toxin’Roll: Electrostatic Charge Repulsion as a Dynamic Venom Resistance Trait in Pythonid Snakes

**DOI:** 10.3390/toxins16040176

**Published:** 2024-04-04

**Authors:** Uthpala Chandrasekara, Emilie M. Broussard, Darin R. Rokyta, Bryan G. Fry

**Affiliations:** 1 Adaptive Biotoxicology Lab, School of the Environment, University of Queensland, St Lucia, QLD 4072, Australia; u.chandrasekara@uq.net.au; 2 Department of Biological Science, Florida State University, 319 Stadium Drive, Tallahassee, FL 32306, USA; ebroussard@bio.fsu.edu (E.M.B.); drokyta@bio.fsu.edu (D.R.R.)

**Keywords:** snake venom, electrostatic charge repulsion, venom resistance, pythons, molecular evolution, predator–prey dynamics

## Abstract

The evolutionary interplay between predator and prey has significantly shaped the development of snake venom, a critical adaptation for subduing prey. This arms race has spurred the diversification of the components of venom and the corresponding emergence of resistance mechanisms in the prey and predators of venomous snakes. Our study investigates the molecular basis of venom resistance in pythons, focusing on electrostatic charge repulsion as a defense against α-neurotoxins binding to the alpha-1 subunit of the postsynaptic nicotinic acetylcholine receptor. Through phylogenetic and bioactivity analyses of orthosteric site sequences from various python species, we explore the prevalence and evolution of amino acid substitutions that confer resistance by electrostatic repulsion, which initially evolved in response to predatory pressure by *Naja* (cobra) species (which occurs across Africa and Asia). The small African species *Python regius* retains the two resistance-conferring lysines (positions 189 and 191) of the ancestral *Python* genus, conferring resistance to sympatric *Naja* venoms. This differed from the giant African species *Python sebae*, which has secondarily lost one of these lysines, potentially due to its rapid growth out of the prey size range of sympatric *Naja* species. In contrast, the two Asian species *Python brongersmai* (small) and *Python bivittatus* (giant) share an identical orthosteric site, which exhibits the highest degree of resistance, attributed to three lysine residues in the orthosteric sites. One of these lysines (at orthosteric position 195) evolved in the last common ancestor of these two species, which may reflect an adaptive response to increased predation pressures from the sympatric α-neurotoxic snake-eating genus *Ophiophagus* (King Cobras) in Asia. All these terrestrial *Python* species, however, were less neurotoxin-susceptible than pythons in other genera which have evolved under different predatory pressure as: the Asian species *Malayopython reticulatus* which is arboreal as neonates and juveniles before rapidly reaching sizes as terrestrial adults too large for sympatric *Ophiophagus* species to consider as prey; and the terrestrial Australian species *Aspidites melanocephalus* which occupies a niche, devoid of selection pressure from α-neurotoxic predatory snakes. Our findings underline the importance of positive selection in the evolution of venom resistance and suggest a complex evolutionary history involving both conserved traits and secondary evolution. This study enhances our understanding of the molecular adaptations that enable pythons to survive in environments laden with venomous threats and offers insights into the ongoing co-evolution between venomous snakes and their prey.

## 1. Introduction

Venom systems are excellent models for studying the evolution of biological adaptations [1,2,3,4]. Venom in snakes is a pivotal trait that originally evolved for the incapacitation of prey [5]. This evolution, driven by the need to overcome the defensive mechanisms of prey species, has resulted in the complex diversification of venom components [6,7]. The selective forces at play are closely linked to the specific physiological systems of the prey, which in turn have spurred the evolution of countermeasures in these species to mitigate the effects of these powerful toxins [8]. This reciprocal progression of adaptation and counteradaptation has led many animal groups that coexist with snakes, and are regularly targeted by them, to undergo substantial positive selection.

Consequently, these prey species have managed not just to endure but to thrive in the presence of venomous predators, illustrating the significant role of natural selection in the evolutionary race between predator and prey [9,10]. The evolutionary advancements that emerge from this perpetual struggle for existence demonstrate the tenacity and versatility of organisms when confronted with life-threatening challenges. Delving into this evolutionary narrative offers a window not only into the core principles of natural selection but also into the delicate equilibrium of ecosystems where predators and prey evolve together, each shaping the other’s survival tactics. Resistance may be accomplished through disparate mechanisms [8]. Two particularly important mechanisms are: the binding by components circulating in the plasma that bind to the toxins and prevent them from reaching their pathophysiological targets [11,12,13]; and a physical change in the target itself resulting in a lower toxin binding affinity [14,15,16,17,18].

This evolutionary drive has led to a marked enhancement of resistance to toxins, such as the convergent evolution to neurotoxin resistance [14,19,20,21,22,23,24,25,26,27,28]. In the evolution of snake venom, the trait of venom peptides targeting and inhibiting neuromuscle signal transduction by attaching to the orthosteric site of the postsynaptic nicotinic acetylcholine receptor (nAChR) α-1 subunit was a key evolutionary innovation [29]. This feature has undergone convergent intensification within two snake families, the Colubridae and Elapidae, with alterations enhancing both the toxicity and selectivity of the neurotoxins [7,30,31,32,33,34,35]. In line with the Red Queen hypothesis [36], a reciprocal development of venom resistance has emerged among both the prey and predators of venomous snakes. α-neurotoxin resistance is accomplished by target modification, attributed to either steric hindrance or electrostatic charge repulsion mechanisms [8].

Steric hindrance arises from structural changes to the orthosteric site of the α-1 subunit of the nAChR. The earliest recognized example of steric hindrance, N-glycosylation, was detected in the Egyptian Mongoose (*Herpestes ichneumon*), a predator of certain cobras [6]. This trait was later identified as a basal trait of the mongoose/meerkat family (Herpestidae), which includes several snake-consuming species [37,38]. This resistance method has been found to have evolved convergently across various taxa that interact with venomous snakes, including within snakes themselves, providing self-immunity against their own venom [37,38,39]. This mutation replaces an amino acid with an asparagine (N) residue (frequently at orthosteric site positions 187 or 189), which is subsequently modified post-translationally to attach a bulky glycosylation side chain. The resultant glycan structure on the asparagine acts as a physical barrier, interfering with the ability of the α-neurotoxins to bind to the receptor and thus conferring resistance. While acetylcholine, the small endogenous neurotransmitter, continues to bind to the orthosteric site, triggering muscle contractions, there appears to be a slight fitness disadvantage, suggested by a secondary loss of resistance in lineages that radiate outside the range of α-neurotoxic predators and are therefore no longer subjected to that predatory selection pressure [38]. Another steric hindrance strategy includes the replacement of proline (P) [20], especially at positions 194 and 197 [26,38,39]. Proline’s unique conformational properties, which induce a tight turn in the peptide chain, are critical for maintaining protein structure. The alterations from proline to different amino acids can induce structural changes in the nAChR, hindering the effective attachment of α-neurotoxins and enhancing neurotoxic venom resistance.

Electrostatic charge repulsion presents an alternate mechanism for countering snake venom, and, like steric hindrance, it has independently evolved in diverse groups, including both the prey (Burmese Python (*Python bivittatus*)) and predators (such as the Honey Badger (*Mellivora capensis*)) of venomous snakes [26,28,37,39,40]. The Honey Badger’s iconic resistance to cobra venom is linked to a singular mutation at the nAChR’s orthosteric site: tryptophan (W) at position 187 replaced by the positively charged arginine (R) [26,40]. The prey species Burmese Python has evolved resistance through the replacement of negatively charged amino acid (aspartic acid (D) or glutamic acid (E)) at positions 191 or 195 in the orthosteric site with a positively charged lysine (K) [26,28,39]. Introducing positive charges on the key receptor sites leads to electrostatic repulsion [26] due to the high concentration of positive charges on the surface of the neurotoxic peptides, which themselves evolved, in response, to facilitate the binding to the negatively charged orthosteric site residues [6]. However, even the elimination of the negativecharge without the replacement by positive charge but with neutral charge, is enough to significantly decrease the binding efficiency of the α-neurotoxins of the snake venom [28].

*P. bivittatus* has been shown to be unique, relative to pythons in other genera, in being resistant to the snake venom α-neurotoxins [26]. However, due to lack of orthosteric site sequence information for other *Python* species it is unclear if this is a trait unique to *P. bivittatus* or if venom resistance is more widespread across this genus. To fill this knowledge gap, we sequenced the orthosteric site from other *Python* species and subsequently undertook bioactivity testing to ascertain the relative resistance to a diversity of α-neurotoxic snake venoms. The results provide insights into the adaptive significance of venom resistance and the evolutionary path that has led to these remarkable biological capabilities.

## 2. Results

**Figure 1:**
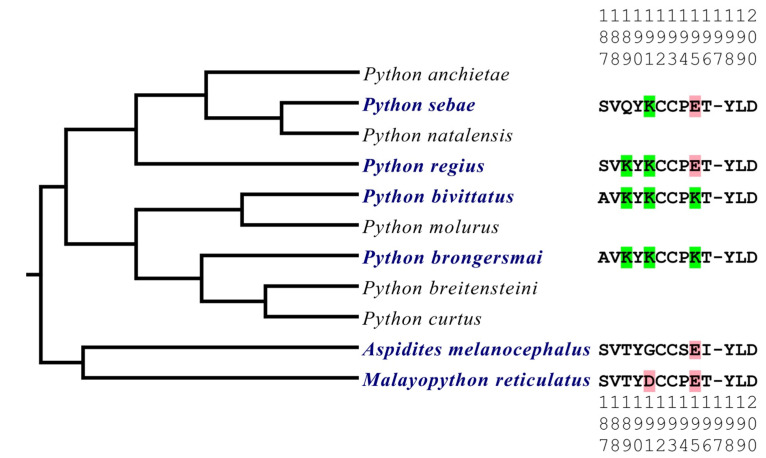
A phylogenetic tree [43] of the species studied (in blue) with additional *Python* species present to give the evolutionary context of the orthosteric site diversifications within this genus. The positively charged amino acid lysine (K) is highlighted in green while the negatively charged amino acids aspartic acid (D) and glutamic acid (E) are highlighted in red. The vertical numbers represent the orthosteric site amino acid positions.

To ascertain the relative influence of geography and morphology, we sequenced an additional giant species (*P. sebae* from Africa) and two small species (*P. regius* from Africa and *P. brongersmai* from Asia) and compared the sequence variations against the giant Asian species *P. bivittatus* (Figure 1). *P. regius* represents the ancestral state within the *Python* genus, possessing two positively charged lysines at positions 189 and 191. This condition was secondarily derived on two occasions, once involving the loss of a lysine (*P. sebae*), once involving the gain of a lysine (*P. bivittatus* and *P. brongersmai*). *P. sebae* has secondarily lost the lysine at 189, retaining only the lysine at position 191. In contrast, at the base of the Asian python radiation, a third lysine has evolved at position 195, with *P. brongersmai* and *P. bivittatus* having identical orthosteric sites. 

It is conspicuous that the two Asian species (*P. bivittatus* and *P. brongersmai*) have the highest levels of positive charges within the orthosteric site, which suggests a stronger predatory pressure. While *Naja* (cobra) species are widespread snake-eating predators in both Africa and Asia, the much larger snake-eating predators in the *Ophiophagus* genus are only found in Asia [41]. The giant species *P. sebae* reaches sizes > 7 m and would therefore soon outgrow the prey size range of <2 m *Naja* species; the equally massive *P. bivittatus* would be in the prey size range of *Ophiophagus* species. For a longer period, which is consistent with the differential presence of lysines in the orthosteric site, with *P. sebae*’s orthosteric site suggestive of the secondary loss of resistance paralleling the evolution of gigantism.

**Figure 2:**
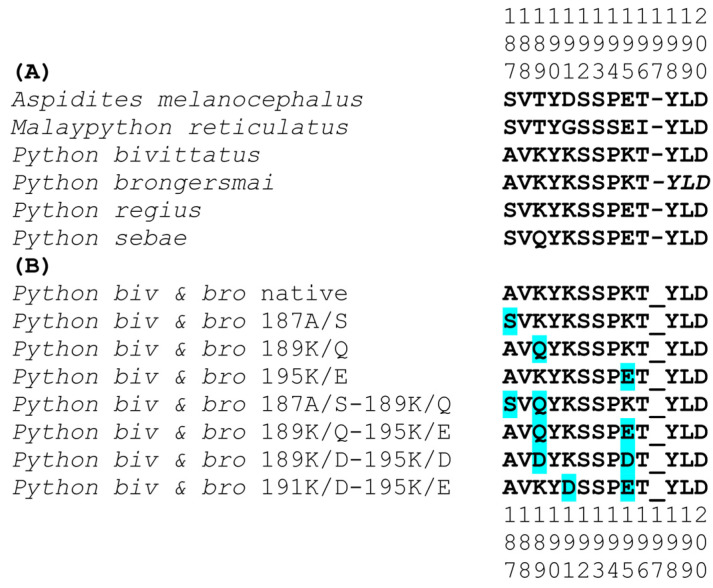
(**A**) Native and (**B**) mutant peptide mimotopes tested with the biolayer interferometry (Figure 3). The cysteine doublet was replaced with a serine doublet during the peptide synthesis to prevent uncontrolled postsynthetic thiol oxidation [44]. In the mutant sequences, the changes are indicated with blue highlights. The vertical numbers represent the orthosteric site amino acid positions.

**Figure 3:**
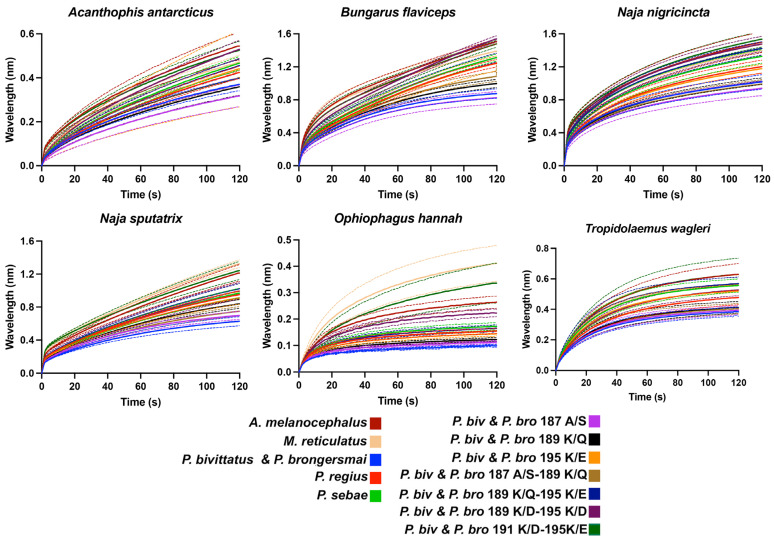
Line graphs of bioactivity testing for binding by a diversity of α-neurotoxic snake venoms using biolayer interferometry immobilized peptide mimotopes corresponding to native and mutant forms (Figure 2). Higher values indicate more venom binding. Area under the curve values are shown in Figure 4.

**Figure 4:**
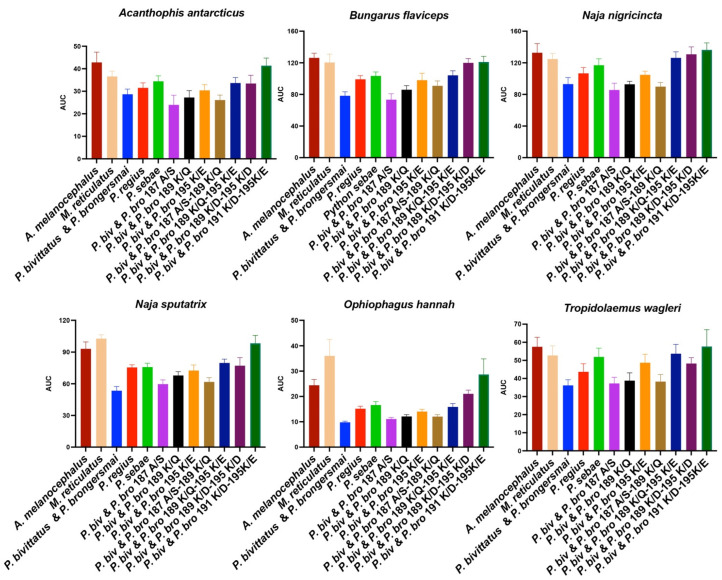
The area under the curve bar graphs of the bioactivity testing for binding by a diversity of α-neurotoxic snake venoms using biolayer interferometry immobilized peptide mimotopes corresponding to native and mutant forms (Figure 2). Higher values indicate more venom binding. The line graph presentations are shown in Figure 3.

To test this hypothesis, using our validated biolayer interferometry protocols [6,26,27,28,42], we constructed peptidic mimotopes corresponding to the orthosteric sites (Figure 2) and tested for the relative binding by a diversity of α-neurotoxic snake venoms (Figure 3 and Figure 4). The relative binding affinity followed the relative presence of lysines:

- *A. melanocephalus* was bound the strongest by all venoms, consistent with this species retaining both the Pythonidae family’s negatively charged amino acids (191D and 195E) and the lack of any positive charges in the orthosteric site.
- *M. reticulatus* was strongly bound but at a level less than *A. melanocephalus*, consistent with it having only one negatively charged amino acid in the orthosteric site (195E) due to the secondary loss of 191D.
- *P. sebae* was bound less strongly than *M. reticulatus* but higher than *P. bivittatus, P. brongersmai,* or *Python regius,* consistent with it retaining only one of the *Python genus* lysine mutations (191K), with 189K replaced by glutamine (Q).
- *P. regius* displayed a lower venom binding affinity than *P. sebae* but bound stronger than *P. bivittatus* and *P. brongersmai*, consistent with it having two lysine mutations (189K and 191K), one more than *P. sebae* but one fewer than *P. bivittatus* and *P. brongersmai.*
- *P. bivittatus* and *P. brongersmai* were the most resistant to binding by any of the venoms, consistent with both species having three lysine mutations (189K, 191K, and 195K).
- A stepwise replacement of the lysine residues [26] confirmed the relative role of the positively charged amino acids in the evolution of venom resistance in *P. bivittatus* and *P. brongersmai.*

## 3. Discussion

The results of our study provide intriguing insights into the molecular evolutionary dynamics of venom resistance among species within the *Python* genus. Our comparative analysis has revealed that the distribution of lysine residues, which confer resistance to α-neurotoxins, varies across the sampled species. The small species *P. regius* represents the ancestral state of the *Python* genus, exhibiting a unique pattern of possessing two positively charged lysines at positions 189 and 191. This suggests that the last common ancestor of the *Python* genus already possessed some level of resistance against α-neurotoxins, in contrast to the lack of this trait in other genera within the Pythonidae family. As such it is predicted that the other small African *Python* species *P. anchietae* will have an orthosteric site similar to *P. regius.* The secondary loss of the lysine residue at position 189 in the giant species *P. sebae*, juxtaposed with its retention of the lysine at position 191, suggests that there is a differential predatory ecology acting upon this species, which parallels the evolution of gigantism, indicative of a ‘use it or lose it’ selection pressure as has been seen in other species [8]. As *P. bivittatus* and *P. brongersmai* are on the same branch of the phylogenetic tree, this indicates the novel lysine at position 195 is a trait inherited from their common ancestor.

Our hypothesis is, therefore, that the two Asian python species *P. bivittatus* and *P. brongersmai* have developed an increased resistance to α-neurotoxins due to stronger predatory pressures, particularly from the genus *Ophiophagus*, which comprises large snake-eating predators exclusive to Asia [41]. In contrast, *P. sebae*, which grows rapidly to sizes beyond the prey range of *Naja* species, might have experienced a secondary loss of resistance. This is likely an adaptation paralleling the evolution of gigantism, as its massive size would be a deterrent to most predators, reducing the selection pressure for venom resistance. Both *P. bivittatus* and *P. brongersmai* are terrestrial, in contrast to the much less resistant *M. reticulatus*, which occupies an arboreal niche as juveniles before reaching sizes as terrestrial adults too massive to be prey for even the largest of the *Ophiophagus* species [45]. In contrast, the terrestrial *A. melanocephalus* occupies a niche in Australia that is not under pressure by α-neurotoxic predatory elapid snakes [46].

The bioactivity testing using the peptidic mimotopes of the orthosteric sites confirmed our predictions, based on lysine presence, of the relative susceptibility to α-neurotoxins. The species with a higher number of lysine residues showed a reduced binding affinity to α-neurotoxic snake venoms, consistent with the previously noted patterns [26], thus affirming the role of these residues in imparting resistance. The gradation of venom binding from *Aspidites melanocephalus*, with the strongest binding due to the retention of the ancestral Pythonidae family’s negatively charged sites, to the highly resistant *P. bivittatus* and *P. brongersmai*, with the lowest venom binding, underscores the adaptive significance of these mutations.

These findings provide a compelling narrative of the interplay between predator–prey dynamics and molecular evolution. The evolution of venom resistance mechanisms in pythons is a testament to the selective pressures exerted by predatory snakes and the resulting arms race that drives the diversification of these defensive traits. Further research in this field will not only elucidate the mechanisms underlying these evolutionary processes but also enhance our understanding of how organisms adapt to their environments and the evolutionary pressures they face.

## 4. Materials and Methods

***DNA extraction from tissue samples***

- DNeasy Blood & Tissue kit (QIAGEN, Carlsbad, CA, USA) was used to isolated the DNA, using the spin column protocol for all species, except for the *P. brongersmai*, whose data was extracted using the E.Z.N.A. Tissue DNA Kit (Omega Bio-tek, Norcross, GA, USA).
- In total, 25 mg of homogenized tissue samples were mixed with a lysis buffer and Proteinase K solution and 56 °C shake-incubated for 3 h. Several centrifugation steps were undertaken followed by washing with wash buffer solutions.
- Prior to the DNA extraction, the tissues were rinsed with 10% of phosphate-buffered saline (PBS) to remove the 70% ethanol preservative.
- Post-elution, the DNA concentration and purity were determined using the Nanodrop 2000 UV–VIS Spectrophotometer (Thermo Fisher Scientific, Waltham, MA, USA).
- The isolated genomic DNA was stored at −20 °C.

***Amplification of orthosteric site sequence of the nAChR α-1 subunit***

- A ~200 base pair range corresponding to chrna1 (muscular nAChR gene) was amplified by locus-specific primer-directed PCR.
- Primers specific for the orthosteric site of the nAChR were designed using the published *Python bivittatus* CHRNA1 sequence XM_007444717.2.
  ○ Python-F = 5′ TGAATAACTACATGCCGAGTGG 3′.
  ○ Python-R = 5′ CGTGGGTAGATAAAATACTAATCC 3′.
- The following were the PCR reaction contents:
  ○ 25 μL of Taq PCR master mix;
  ○ 3 μL of each primer (10 μM);
  ○ 500 ng of DNA;
  ○ PCR water to adjust to the 50 μL total PCR reaction volume.
- The PCR reaction conditions were as follows:
  ○ Initial denaturation at 95 °C for 3 min (all subsequent denaturation steps were at 95 °C for 30 s);
  ○ Annealing was at 55 °C for 30 s;
  ○ Extension was at 72 °C for 1 min;
  ○ The PCR steps of denaturation, annealing, and extension were repeated for 35 cycles;
  ○ Final extension at 72 °C for 10 min.

***Sequencing of the nAChR***

- Sequencing of the primer-directed locus-specific amplified PCR products was undertaken at the Australian Genome Research Facility, University of Queensland, Australia, and Florida State University’s Core Facilities DNA Sequencing Laboratory, Tallahassee, Florida, using the automated dideoxy sequencing method dual-direction sequencing.
- The sequence reads were aligned and manually curated using the Aliview v.1.1 software (alignment viewer and editor) and Expasy (translate tool) to ascertain the relative absence or presence of the resistance elements in the ligand binding domain of the α1subunit of the nAChR of each of the python species tested.

***Venom stock collection and preparation***

- The venom samples were sourced from the long-term cryogenic collection of the Adaptive Biotoxicology Lab, University of Queensland, St Lucia, Australia.
- All the venom study protocols of this work were performed with the University of Queensland Biosafety Approval #IBC134BSBS2015 and the University of Queensland Animal Ethics Approval 2021/AE000075.
- The lyophilized crude venom samples were reconstituted with double-deionized water (ddH_2_O) before use. The centrifugation was performed at 14,000 RCF for 10 min with a temperature of 4 °C.
- Subsequently, the pellet (if any) was discarded, and the supernatant was used to make a working venom stock of 1 mg/mL in 50% of glycerol to preserve the enzymatic action while avoiding freezing upon storage at −20 °C.
- The concentrations of the prepared venom stocks were checked at 280 nm with a NanoDrop 2000 UV–VIS Spectrophotometer (Thermo Fisher Scientific, Waltham, MA, USA).

***Mimotope design and preparation***

- 14-amino-acid-longshort peptide mimotopes corresponding to the orthosteric site of the python muscle-type nAChR α-1 subunit were synthesized [6,26,27,28,42].
- Uncontrollable postsynthetic thiol oxidation was prevented by the synthetic peptides having a serine doublet in place of the cysteine doublet [44].
- The mimotopes were dissolved in 100% of dimethyl sulfoxide (DMSO) followed by a 1:10 dilution with double-deionized water in order to make 50 µg/mL of working stocks.
- All prepared mimotope stock solutions were stored at −20 °C for future use.

***Biolayer interferometry assay (BLI)***

- An Octet HTX biolayer interferometry assay was used to measure the neurotoxin receptor binding affinities, following previously published protocols just as with the data acquisition, processing, and statistical analyses [6,27,28,42,47,48,49,50,51].

## Data Availability

The data supporting the reported results can be found in the Supplementary Material.

## Supplementary Materials

The following supporting datasheets can be downloaded at https://www.mdpi.com/article/10.3390/toxins16040176/s1: raw data for Figure 3 line graphs; and raw data for Figure 4 bar graphs.
